# Statistical ecology comes of age

**DOI:** 10.1098/rsbl.2014.0698

**Published:** 2014-12

**Authors:** Olivier Gimenez, Stephen T. Buckland, Byron J. T. Morgan, Nicolas Bez, Sophie Bertrand, Rémi Choquet, Stéphane Dray, Marie-Pierre Etienne, Rachel Fewster, Frédéric Gosselin, Bastien Mérigot, Pascal Monestiez, Juan M. Morales, Frédéric Mortier, François Munoz, Otso Ovaskainen, Sandrine Pavoine, Roger Pradel, Frank M. Schurr, Len Thomas, Wilfried Thuiller, Verena Trenkel, Perry de Valpine, Eric Rexstad

**Affiliations:** 1 CEFE UMR 5175, CNRS, Université de Montpellier, Université Paul-Valéry Montpellier, EPHE, 1919 Route de Mende, 34293 Montpellier Cedex 5, France; 2 Centre for Research into Ecological and Environmental Modelling, University of St Andrews, St Andrews KY16 9LZ, UK; 3 School of Mathematics, Statistics and Actuarial Science, University of Kent, Canterbury, Kent CT2 7NF, UK; 4 IRD, UMR EME 212, Sète, France; 5 Université de Lyon, F-69000, Lyon; Université Lyon 1; CNRS, UMR5558, Laboratoire de 18 Biométrie et Biologie Evolutive, F-69622, Villeurbanne, France; 6 AgroParisTech, UMR MIA 518, Paris, France; 7 Department of Statistics, University of Auckland, Private Bag 92019, Auckland, New Zealand; 8 Irstea, UR EFNO, Centre de Nogent-sur-Vernisson, 45290 Nogent-sur-Vernisson, France; 9 Université Montpellier 2, UMR EME 212, Sète, France; 10 INRA, BioSP, Avignon, France; 11 Laboratorio Ecotono, CRUB, INIBIOMA-CONICET, Bariloche, Argentina; 12 UPR Bsef, CIRAD, Montpellier, France; 13 UM2, UMR AMAP, Bd de la Lironde, TA A-51/PS2, 34398 Montpellier Cedex 5, France; 14 Department of Biosciences, University of Helsinki, Helsinki, Finland; 15 UMR 7204 CNRS UPMC, Centre for Ecology and Conservation Sciences, Muséum National d'Histoire Naturelle, 55-61 rue Buffon, 75005 Paris, France; 16 Mathematical Ecology Research Group, Department of Zoology, University of Oxford, Oxford OX1 3PS, UK; 17 Institute of Landscape and Plant Ecology, University of Hohenheim, 70593 Stuttgart, Germany; 18 Laboratoire d'Ecologie Alpine, UMR CNRS 5553, Université Joseph Fourier, Grenoble I, BP 53, 38041 Grenoble Cedex 9, France; 19 Ifremer, Rue de l’île d'Yeu, BP 21105, 44311 Nantes Cedex 3, France; 20 Environmental Science, Policy and Management, University of California, Berkeley, CA 94720, USA

**Keywords:** citizen science, hidden Markov model, hierarchical model, movement ecology, software package, spatially explicit capture–recapture, species distribution modelling, state–space model

## Abstract

The desire to predict the consequences of global environmental change has been the driver towards more realistic models embracing the variability and uncertainties inherent in ecology. Statistical ecology has gelled over the past decade as a discipline that moves away from describing patterns towards modelling the ecological processes that generate these patterns. Following the fourth International Statistical Ecology Conference (1–4 July 2014) in Montpellier, France, we analyse current trends in statistical ecology. Important advances in the analysis of individual movement, and in the modelling of population dynamics and species distributions, are made possible by the increasing use of hierarchical and hidden process models. Exciting research perspectives include the development of methods to interpret citizen science data and of efficient, flexible computational algorithms for model fitting. Statistical ecology has come of age: it now provides a general and mathematically rigorous framework linking ecological theory and empirical data.

## 1. Introduction

Variability is challenging ecology, from genes to individuals, species or ecosystems: quantifying and explaining biological variation is an ever-important goal. Variability arises from both ecological processes and sampling, requiring the modelling of uncertainty, the very nature of statistics [1,2].

Statistics has long permeated the field of ecology through the contributions of eminent scientists such as Fisher, Haldane and Leslie. However, we detect a recent rise in statistical awareness, manifested in various ways. First, research centres especially devoted to statistical ecology have been created in the USA (Statistical and Applied Mathematical Sciences Institute) and the UK (National Centre for Statistical Ecology). There are also institutes focused on synthesis (e.g. the National Center for Ecological Analysis and Synthesis and the National Institute for Mathematical and Biological Synthesis, both in the USA). Second, new journals dedicated to methodological advances (not only statistical) have been created and are now having considerable impact (notably *Molecular Ecology Resources* and *Methods in Ecology and Evolution*). Third, there are more specialized conferences that provide the opportunity for statisticians to interact with ecologists for mutual benefit. The reasons for this recent rise of statistical ecology are manifold and include the societal demand for scientists to address pressing issues such as global change and the current biodiversity crisis, the need to analyse the massive datasets and the novel data types generated by new technologies, and the popularization of methods through free statistical packages and the increase in computing power. We view the rise of statistical ecology as a sign that ecological and statistical modelling are coming together with the common goal of understanding complex processes in a formal inferential framework for better predictive capabilities. We acknowledge that not all ecologists agree that ecology lends itself to theorization and prediction [3] or that process-based methods necessarily have higher predictive ability than phenomenological models [4,5]. However, past disappointments may simply be due to inappropriate and coarse modelling. If so, progress in both ecological theory and statistical ecology and a better integration of the two should enhance our understanding and our ability to predict ecological phenomena. In the following, we highlight recent trends in statistical ecology and provide perspectives for the future development of this discipline (see also [6]).

We analysed the contents of the abstracts of four International Statistical Ecology Conferences (ISECs) held biannually between 2008 and 2014 to provide a picture of recent trends in statistical ecology (electronic supplementary material, Appendix S1). The quantitative results of this analysis show a temporal shift across the different ISECs, from studies focusing on sampling design issues towards predictive studies that aim to integrate the modelling of processes with the analysis of ecological patterns. These results are further synthesized below.

## 2. Questions being addressed

### (a) Assessing species distribution

Species distribution models (SDMs) are now common tools to investigate the main drivers of species range and to forecast potential impacts of environmental changes on biodiversity. Important innovations include the use of point processes to fit SDMs to presence-only data and the mathematical equivalence of MAXENT (a common SDM tool) to generalized linear models in this context [7]. SDMs are also being extended to several species to improve the model parametrization for rare species and to enable the estimation of co-occurrence patterns. Last, the development of hierarchical occupancy models, with their ability to handle spatial dependence and imperfect detection, paves the way for better modelling of the underlying sources of uncertainty [8].

### (b) Measuring biodiversity

Biodiversity is multifaceted, involving aspects of species richness, functions, traits and phylogeny. Consequently, the choice of relevant diversity indices is challenging, especially when analysing aspects of functional or phylogenetic diversity and when evaluating the dissimilarities among locations (quadrats, sites or regions). Moreover, the potential factors driving the dynamics of biodiversity (e.g. competition and environmental filters) need to be disentangled.

### (c) Investigating population dynamics

In the ISECs, estimation of population size has been a major focus, notably through refinements of capture–recapture (CR) methods. There has been an increase in non-invasive methods that use natural identifying characteristics of animals (camera or acoustic traps, genetic markers), with treatment of misidentification error. In parallel, spatially explicit models have been developed to fully exploit the spatial information in CR data [9,10].

### (d) Understanding animal movements

Movement ecology has shifted from phenomenological models of observable patterns to mechanistic models characterizing the underlying processes. In particular, the use of state–space models that account explicitly for the observation process has now become standard [11], and hierarchical models have been developed to model individual movements as functions of behavioural states, past experiences and environmental heterogeneity [12]. While earlier work relied on discrete-time correlated random walks, the use of continuous-time models and the integration of other types of data (e.g. species interactions and population dynamics) are increasing.

### (e) Interpreting citizen science data

Data from citizen science programmes represent an opportunity to sample large regions and inform long-term monitoring studies. Difficulties arise with recent programmes based on web- and smartphone-based technologies that are characterized by the free participation of many laypersons, loose sampling protocols and heterogeneities in the spatio-temporal distribution of observations. These potential sources of bias may be accounted for by the joint modelling of the ecological and observation processes through, for example, hidden process models [13].

## 3. Material and methods

### (a) Hidden process modelling

Ecologists have broadly adopted hierarchical, state–space and hidden Markov models to deal with the way in which individuals and populations distribute in space and change over time [14]. This reflects a move away from modelling spatio-temporal patterns *per se* and towards modelling the ecological processes that generate those patterns. The timescale of interest might be short, such as for animal behaviour, medium, such as migration and demographic processes, or long, such as for changes in species ranges, composition and biodiversity, or for evolutionary processes. By modelling the underlying processes while accounting for observation error and model uncertainty, we seek to gain in predictive ability and hence in the effectiveness of management actions, whether we are managing a commercial fishery, conserving a threatened population, assessing the impact on biodiversity of habitat loss, predicting response of populations to disturbance or evaluating the effects of climate change on communities.

### (b) Coexistence of frequentist and Bayesian frameworks

Bayesian methods are now widely used, largely because they can more easily accommodate realistic ecological models. However, two notable trends are emerging: an increasing interest in critically evaluating the performance of Bayesian methods from a frequentist perspective [15], and the increasing practicality of frequentist tools for hierarchical models previously only amenable to Bayesian methods (e.g. [16]).

### (c) Dynamic models

Current research in population dynamics addresses the limits of statistical inference and predictions for nonlinear dynamics (e.g. [17]). Beyond the population, dynamic statistical models are now applied at larger spatial and organizational scales to describe the dynamics of species ranges, communities and ecosystem processes (e.g. [18]). A common feature of these recent statistical models is that they describe how large-scale dynamics arise from underlying principles of demography and/or ecophysiology, aiming to base inference and prediction on processes rather than correlations.

### (d) Integrated modelling

Another trend is the popularization of integrated modelling—i.e. combining different datasets in a single, coherent analysis [19]—to address a wide variety of ecological questions. Current developments deal with the issues of goodness-of-fit testing, model selection, integration of recent developments in demography (e.g. integral projection models) and testing the assumption that data from different sources can be considered independent. From an ecological viewpoint, integrated modelling now scales from populations up to communities [20].

## 4. Implementation

### (a) Computational algorithms

The development of efficient and flexible computational algorithms for complex models and big datasets ([integrated nested] Laplace approximations, Hamiltonian Monte Carlo and standard Markov chain Monte Carlo algorithms) requires tremendous research efforts, as does their implementation in software packages (e.g. R-INLA (http://www.r-inla.org/), AD Model Builder (http://admb-project.org/), LaplacesDemon (http://www.bayesian-inference.com/software), Stan (http://mc-stan.org/), Nimble (http://r-nimble.org/), OpenBUGS (http://www.openbugs.net/w/FrontPage), JAGS (http://mcmc-jags.sourceforge.net/), PyMC (http://pymc-devs.github.io/pymc/), MCMCglmm (http://cran.r-project.org/web/packages/MCMCglmm/index.html)). When a complete likelihood cannot be easily calculated, methods for estimation based only on simulations and summary statistics (Synthetic likelihood [21]; Approximate Bayesian Computation [22]) are also receiving attention.

### (b) Software development and evaluation

There is a tension between devoting time to developing new methodology and enabling other researchers to implement it. Although it is easy to self-publish an R package or a graphical user interface (GUI), a culture shift is needed towards more thorough testing and verification of published software. We welcome the initiative of ecological journals to publish software papers, which ensures that publicly available software is peer-reviewed, and endows software development efforts with much-needed professional recognition.

## 5. Advice to statistical ecologists

### (a) Avoiding statistical machismo^1^

Given methodological developments and increasing computing power, there is a great temptation to increase model complexity. In some cases, this is helpful: previously restrictive assumptions about the observation process can be relaxed; previously intractable ecological mechanisms can be expressed as mathematical models and incorporated in estimation. In other cases, however, increasing complication can lead to less robust inference or ecologically insignificant improvements, which nevertheless waste practitioners' time and direct their energies away from less glamorous topics such as improved data collection; there is also often an increased chance of mistakes in implementation. There is a clear need for an evaluation strategy of new, often complex statistical methods to determine the scope of beneficial application for ecology [23]. *Beneficial* means that for a given ecological question and dataset, applying the new or modified method provides clearer results and avoids drawing flawed conclusions. Comprehensive model evaluation must include consideration of sample design, covariate selection, goodness-of-fit and parameter redundancy diagnostics.

### (b) Going one step further

Many ecological applications are motivated by scientific support for conservation or management decisions. Statistical decision theory has much to offer, both directly in terms of helping rational decision-making, and also in optimizing future data-collection efforts.

## 6. Conclusion

The dialogue between statisticians and ecologists has intensified over recent decades, and ISECs have contributed to this dialogue. We encourage even more mixing between statisticians and ecologists, by exhorting the former to go to the field to gain a sound understanding of the data for relevant modelling [24], and the latter to embrace courses in mathematics that underpin the reliable application of statistical methods [25].

In summary, the statistical approaches developed for ecology are maturing towards a statistically rigorous, explanatory and possibly predictive framework for linking theory, data and applications. Exciting research directions are ahead of us that we hope will help to address pressing issues in the context of global change.

## Supplementary Material

Text analysis of the ISEC abstracts

## Supplementary Material

R code

## Supplementary Material

data file #1

## Supplementary Material

data file #2

## Endnote

1 See http://dynamicecology.wordpress.com/2012/09/11/statisticalmachismo/.
