# Supplementary material for: Statistical ecology comes of age
Source: Biol Lett. 2014 Dec;10(12):20140698. doi: 10.1098/rsbl.2014.0698 (PMC4298184; doi:10.1098/rsbl.2014.0698)
Supplement: Text analysis of the ISEC abstracts [file rsbl20140698supp1.docx]

**Appendix 1**.

The R code to perform the analyses described below is provided in the script ISEC-analysis.R as well as the data which are in the ISECData.rda and ISECGraph.rda files.

We performed a text mining analysis and analyzed the lists of the 25 most common words in each ISEC abstract volume (Figure A1). The word *dynam* appeared in the 2014 list, *chang* and *process* in 2012 and 2014 and *time* in 2010, 2012 and 2014 suggesting a growing interest in integrating mechanisms to explain ecological patterns in time. *Predict* reached the top 25 list in 2014 and *chang* in 2012 and 2014, which is in line with a rising concern for global change and with related efforts to predict ecological dynamics under environmental change. The words *distance*, *survey*, *density* and *design* disappeared from the list after ISEC 2008. This reflects the main focus of the first conference on sampling design issues while ISECs 2010, 2012 and 2014 reflected a wider range of interests (e.g., *movement ecolog*y appeared in 2012).

Now focusing on the 16 words that are common to the abstracts of all four ISECs (Figure A1), statistical ecology is without surprise about fitting *model*s to *data* to *estim*ate *parameter*s of ecological relevance. This is achieved by developing *method*s to determine the main *effect*s explaining the *differ*ent patterns in the *distribution*s *of* *individu*als, *popul*ations and *speci*es. The quantity of interest is predominantly the *abund*ance of *anim*als, considered at different *spatial* scales with a particular attention to the issue of imperfect *detect*ion and adequate *sampl*ing scheme.


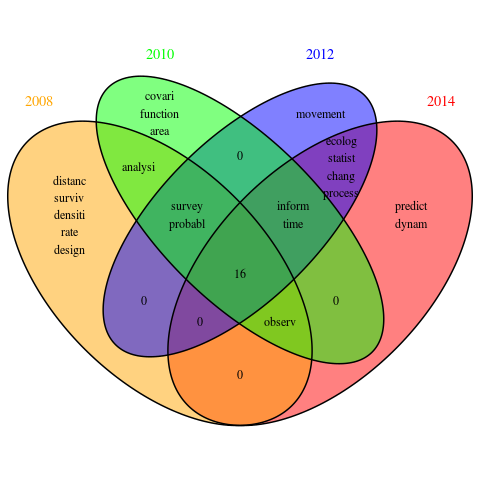


**Figure A1**. The 25 most frequent words in the abstracts of each ISEC using a Venn diagram. We found 16 words (or at least their root) common to all ISECs: abund, anim, data, detect, differ, distribution, effect, estim, individu, method, model, paramet, popul, sampl, spatial, speci. See Table A1 for the full list of 25 words per year.

**Table A1**. The 25 most frequent words in the ISEC abstracts (sorted by the number of occurrences). The terms common to all ISEC editions are in bold.

| 2008 | 2010 | 2012 | 2014 |
| --- | --- | --- | --- |
| **model** | **model** | **model** | **model** |
| **estim** | **estim** | **data** | **data** |
| **data** | **data** | **estim** | **speci** |
| **popul** | **speci** | **popul** | **estim** |
| **speci** | **popul** | **speci** | **popul** |
| **sampl** | **method** | **spatial** | **method** |
| **method** | **sampl** | **method** | **distribut** |
| **abund** | **abund** | **individu** | **spatial** |
| survey | **differ** | **sampl** | **sampl** |
| **spatial** | **detect** | **paramet** | ecolog |
| **detect** | **spatial** | time | **differ** |
| probabl | survey | **distribut** | **abund** |
| **paramet** | observ | survey | **individu** |
| **anim** | inform | **abund** | time |
| **individu** | **paramet** | **effect** | **detect** |
| analysi | **anim** | **differ** | **paramet** |
| distanc | **effect** | **detect** | chang |
| **distribut** | **individu** | inform | observ |
| surviv | analysi | ecolog | predict |
| observ | probabl | probabl | process |
| densiti | **distribut** | **anim** | dynam |
| **differ** | time | movement | **effect** |
| rate | covari | statist | **anim** |
| design | function | chang | statist |
| **effect** | area | process | inform |

In addition, we performed a multivariate analysis (non-symmetric correspondence analysis) of the 50 most common words found in the abstracts of the four conferences (Figure A2). Figure A2a shows major trends of semantic variation among the abstracts. The first major trend (abscissa) contrasts the studies focusing on sampling design issues (e.g., high positive scores of *transect*, *design*, *sample*, *detec*) and the studies focusing on characterizing processes and resulting patterns (negative scores). The second axis contrasts the field of population studies based on capture-recapture approaches vs. approaches investigating community dynamics, habitat modelling and species distributions (e.g., high positive scores of *communiti*, *speci*, *habitat*, *distribut*). These two axes represent 10.04% of the overall variation among abstracts. Figure A2b shows the 90% convex hulls of each ISEC conference based on the scores of their abstracts. We found a significant variation (randomization test, p < 0.001) with an overall trajectory toward lower scores on the first axis (more process-oriented works) and toward more emphasis on community dynamics, habitat modelling and species distributions on the second axis.


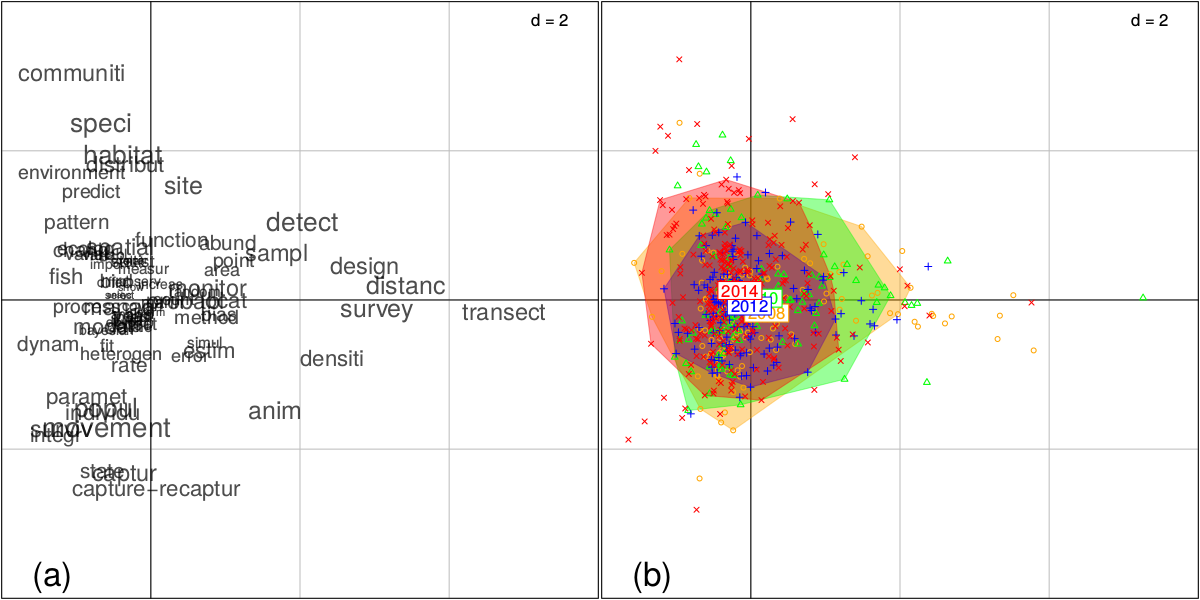


**Figure A2**. Semantic variations found among the abstracts of the four ISECs identified by a non-symmetric correspondence analysis of the word-by-abstract table. The resulting first factorial map shown here illustrates major trends of semantic variation among the abstracts. (a) The higher scoring words are those most contributing to semantic variation in the factorial map. The size of labels is proportional to the contribution of each word to the first two axes. (b) Distinguishing the 90% convex hulls of abstracts for the four ISECs underlines thematic variations through time.

We also addressed the structure of the research fellow communities participating in ISECs. The co-authorship network was built and analyzed using a stochastic block model (Figure A3) to identify groups of authors. Based on the Integrated Completed Likelihood criterion, 15 groups of authors were detected. The isolated contributors are grouped in cluster 9, which is one of the most important in numbers. The other groups may be named communities since they were characterized by high within probability connectivity. Cluster 1 (in dark blue) was found to be a central hub of in the ISEC community. A meta-community, formed by clusters 1, 7, 10, 12 and 15 was identified and mainly contained the initial ISEC contributors. The 9 remaining clusters exhibited high level of within-connections but poor between-connections. This may indicate a need for more communications and exchanges between communities and disciplines within statistical ecology.


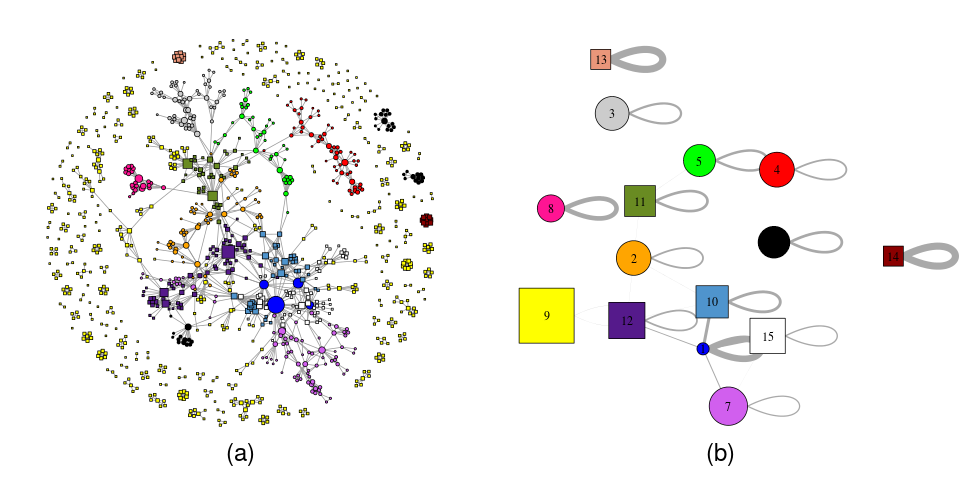
**Figure A3**. Analysis of the ISEC coauthorship network. The first graph (a) presents the network of copublications, based on the talks given during the four ISEC editions. Each vertex represents a contributor. The colors indicate the clusters detected by the stochastic block model analysis. The size of a vertex is proportional (in log scale) to the number of coauthors. The second figure (b) sums up the properties of each cluster. The width of the edges depends on the probabilities of connection between or within clusters, while the size of the vertices relies on the assignment marginal probabilities.
